# Detoxification of methylglyoxal by the glyoxalase system is required for glutathione availability and virulence activation in *Listeria monocytogenes*

**DOI:** 10.1371/journal.ppat.1009819

**Published:** 2021-08-18

**Authors:** Andrea Anaya-Sanchez, Ying Feng, John C. Berude, Daniel A. Portnoy

**Affiliations:** 1 Graduate Group in Microbiology, University of California, Berkeley, Berkeley, California, United States of America; 2 Department of Molecular and Cell Biology, University of California, Berkeley, Berkeley, California, United States of America; 3 Department of Plant and Microbial Biology, University of California, Berkeley, Berkeley, California, United States of America; University of Texas Medical School at Houston, UNITED STATES

## Abstract

*Listeria monocytogenes* is a Gram-positive, food-borne pathogen that lives a biphasic lifestyle, cycling between the environment and as a facultative intracellular pathogen of mammals. Upon entry into host cells, *L*. *monocytogenes* upregulates expression of glutathione synthase (GshF) and its product, glutathione (GSH), which is an allosteric activator of the master virulence regulator PrfA. Although *gshF* mutants are highly attenuated for virulence in mice and form very small plaques in host cell monolayers, these virulence defects can be fully rescued by mutations that lock PrfA in its active conformation, referred to as PrfA*. While PrfA activation can be recapitulated *in vitro* by the addition of reducing agents, the precise biological cue(s) experienced by *L*. *monocytogenes* that lead to PrfA activation are not known. Here we performed a genetic screen to identify additional small-plaque mutants that were rescued by PrfA* and identified *gloA*, which encodes glyoxalase A, a component of a GSH-dependent methylglyoxal (MG) detoxification system. MG is a toxic byproduct of metabolism produced by both the host and pathogen, which if accumulated, causes DNA damage and protein glycation. As a facultative intracellular pathogen, *L*. *monocytogenes* must protect itself from MG produced by its own metabolic processes and that of its host. We report that *gloA* mutants grow normally in broth, are sensitive to exogenous MG and severely attenuated upon IV infection in mice, but are fully rescued for virulence in a PrfA* background. We demonstrate that transcriptional activation of *gshF* increased upon MG challenge *in vitro*, and while this resulted in higher levels of GSH for wild-type *L*. *monocytogenes*, the glyoxalase mutants had decreased levels of GSH, presumably due to the accumulation of the GSH-MG hemithioacetal adduct. These data suggest that MG acts as a host cue that leads to GSH production and activation of PrfA.

## Author summary

*Listeria monocytogenes* is a facultative intracellular pathogen that dramatically changes gene expression upon infection of host cells by activating its major virulence regulator PrfA. The allosteric activator of PrfA is glutathione (GSH) that is produced by the bacterial glutathione synthase GshF. The requirement for GshF can be bypassed by PrfA mutations (PrfA*) that are locked in their active form. In this study we screened for additional mutations that prevented virulence gene expression but were rescued by PrfA*. One of these mutations was in *gloA*, which encodes glyoxylase A, which detoxifies methylglyoxal (MG) in a glutathione-dependent fashion. In response to MG, *gloA* mutants up-regulated *gshF* mRNA but have lower levels of available GSH. These and other data suggest that MG production by the host activates *gshF* expression leading to elevated GSH levels and increased virulence gene expression.

## Introduction

*Listeria monocytogenes* is a Gram-positive facultative intracellular pathogen and a significant cause of human disease, but is also an excellent model system to study basic aspects of host-pathogen interactions [1]. *L*. *monocytogenes* is an ubiquitous saprophyte found in soil, water and vegetation, and can contaminate a variety of food products leading to periodic outbreaks of disease [1,2]. Following ingestion by susceptible mammalian hosts, *L*. *monocytogenes* transitions into an intracellular pathogen largely by the transcriptional up-regulation of virulence gene expression [2,3].

In order to access its replicative niche in the host cytosol, *L*. *monocytogenes* secretes listeriolysin O (LLO), a pore-forming, cholesterol-dependent cytolysin that mediates disruption of the phagosome [3]. In the cytosol, *L*. *monocytogenes* up-regulates the expression and synthesis of the master virulence regulator PrfA, that directly regulates the transcription of nine virulence genes including ActA, a surface protein that mediates host actin polymerization and promotes movement and dissemination into neighboring cells [4]. PrfA is a member of the cAMP receptor protein (Crp) family of transcription factors that are allosterically regulated by small-molecule activators. Transcriptional activation of PrfA requires allosteric binding to glutathione (GSH) that is produced by *L*. *monocytogenes* glutathione synthase (GshF) [5]. *L*. *monocytogenes* strains that lack *gshF* are attenuated for virulence, but the requirement for glutathione can be bypassed by mutations that lock PrfA on its active conformation and are referred to as PrfA* [5].

Although there is a comprehensive understanding of the intracellular life cycle of *L*. *monocytogenes*, less is known about the exact biological cue(s) sensed by this pathogen that drive the transition from extracellular to intracellular gene expression. Previous studies have shown PrfA activation *in vitro* by the addition of reducing agents [6], however, what *L*. *monocytogenes* senses *in vivo* remains unknown. Nonetheless, since GSH serves as an antioxidant and *gshF* is upregulated *in vivo*, we speculate that reactive oxygen species (ROS), reactive electrophilic species (RES) and/or reactive nitrogen species (RNS) may be inflicting redox stress leading to upregulation of GshF.

**Fig 1:**
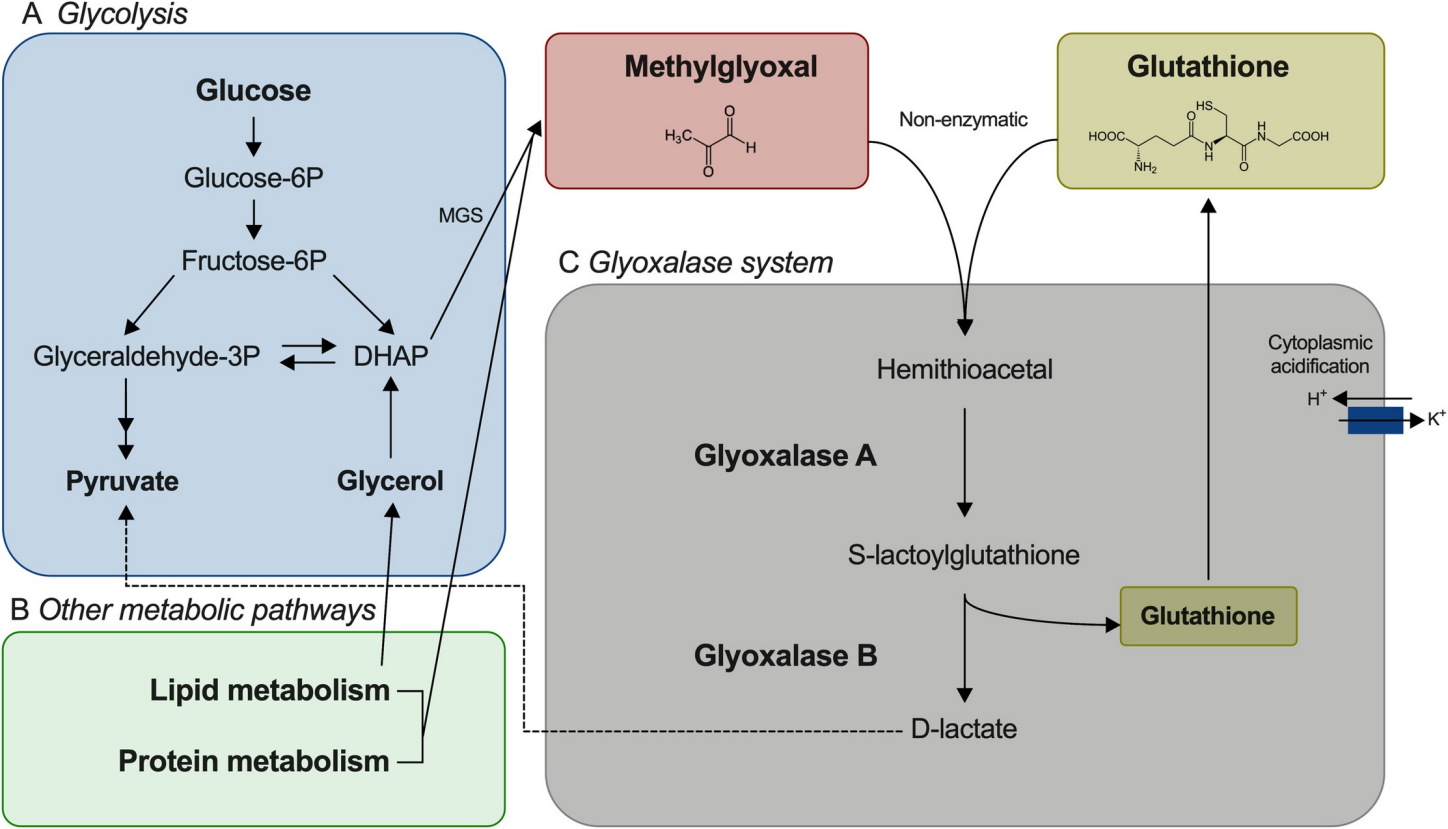
Methylglyoxal production and detoxification pathways. **(**A) MG is formed mainly as a byproduct of glycolysis. In most organisms it is transformed from dihydroxyacetone phosphate (DHAP) by methylglyoxal synthase (MGS). (B) MG production can also result from the metabolism of lipids and proteins. (C) MG detoxification occurs mainly through the glyoxalase system that consists of two enzymes glyoxalase A (GloA) and glyoxalase B (GloB). MG can react non-enzymatically with reduced glutathione (GSH) to form a hemithioacetal which is transformed to S-lactoylglutathione by GloA. S-lactoylglutathione activates potassium efflux pumps that acidify the cytoplasm and confer protection against MG. GloB then transforms S-lactoylglutathione to D-lactate, recycling GSH in the process. Adapted from Allaman *et al*, 2015 [7].

Methylglyoxal (MG) is a RES that is an ubiquitous byproduct of cellular metabolism and is produced by both bacteria and host cells [7,8]. MG reacts with arginine, lysine, and cysteine residues in proteins, resulting in the formation of advanced glycation end products (AGEs), which often leads to protein inactivation [7,9]. Additionally, MG modifies guanine bases, resulting in DNA damage and increased mutation rates [10,11]. This toxic metabolite can be synthesized through enzymatic (methylglyoxal synthase) and non-enzymatic reactions [12]. While MG production can result from protein and lipid metabolism (Fig 1B), glycolysis is the most important source of endogenous MG (Fig 1A) [7]. Of note, *Mycobacterium tuberculosis* infected macrophages secrete up to 1.6 mM MG into culture media [13]. As a facultative intracellular pathogen, *L*. *monocytogenes* must protect itself from both endogenously produced MG and that produced by its host.

The major mechanism of protection from MG is mediated by the glyoxalase system (Fig 1C). Briefly, MG detoxification is initiated by the non-enzymatic reaction of this toxic metabolite with a low-molecular-weight thiol such as reduced glutathione (GSH) or bacillithiol, forming a hemithioacetal. This molecule is then transformed to S-lactoylglutathione by glyoxalase A (GloA). In *E*. *coli* and *B*. *subtilis*, the intermediate generated by GloA activates potassium efflux pumps that acidify the cytoplasm and confer protection against MG-induced DNA damage [10,14]. S-lactoylglutathione is converted to D-lactate by glyoxalase B (GloB), recycling GSH in the process [15].

In order to better understand the biological cues sensed by *L*. *monocytogenes* upon entry into the host cytosol, we screened for small-plaque mutants that were rescued in a PrfA* background. This screen identified glyoxalase A, the first enzyme in the glyoxalase system (Fig 1C). *L*. *monocytogenes gloA* mutants were attenuated for plaque formation and in an IV mice model of infection. Virulence defects were fully rescued in a PrfA* background. The presence of MG increased *gshF* mRNA and GSH levels *in vitro*, resulting in the activation of *actA* transcription. These data suggest that MG acts as a biological cue that leads to PrfA activation.

## Results

### A genetic screen identifies *gloA* as a small-plaque mutant rescued by PrfA*

We sought to identify genes other than *gshF* involved in *L*. *monocytogenes* virulence activation. *L*. *monocytogenes* is capable of spreading to neighboring cells, which is evidenced by its capacity to form plaques in tissue culture cell monolayers. The plaque assay is a straightforward and efficient method to screen for mutants defective in intracellular growth or cell-to-cell spread [16]. A *himar-1* transposon library was used to screen for transposon insertions that caused a small-plaque phenotype in the L2 murine fibroblast cell line after 3 days of infection. A total of approximately 100,000 plaques were screened, 300 were initially picked, and 132 were further selected after the purification cycles, representing 34 genes. The selected small-plaque mutants had at least a 20% decrease in plaque size compared to wild-type *L*. *monocytogenes*. Transposon insertions were transduced into wild-type and PrfA* backgrounds and compared using the plaque assay (Table 1). As the goal of the genetic screen was to better understand the cytosolic biological cues leading to PrfA activation, we were primarily interested in transposon mutations that could be rescued by PrfA*, which would suggest a role in *L*. *monocytogenes* virulence activation. As expected, insertions in *gshF* fulfilled this criteria [5], but we also identified an insertion in *lmo2168* that was rescued in a PrfA* background to within 10% of the plaque size of wild-type PrfA* (Table 1). *GloA (lmo2168)* encodes glyoxalase A, also known as lactoylglutathione lyase, an enzyme required for MG detoxification (Fig 1).

**Table 1:** Genes identified in small-plaque screen.

Lmo number	LMRG number	# Hits^a^	Annotation	Background	Plaque size^b^	Background	Plaque size^b^
			10403S	wt	100 ± 1.20	PrfA*	109 ± 1.46
**0201**	**02623**	4	*plcA*	wt	24 ± 0.58	PrfA*	38 ± 0.84
**0202**	**02624**	2	*hly*	wt	36 ± 1.56	PrfA*	34 ± 0.69
**0203**	**02625**	6	*mpl*	wt	23 ± 0.78	PrfA*	48 ±1.31
**0205**	**02627**	6	*plcB*	wt	42 ± 1.21	PrfA*	27 ± 1.12
**0402**	**00095**	1	hypothetical protein	wt	81 ± 1.27	PrfA*	90 ± 1.04
**0898**	**02322**	1	hypothetical protein	wt	74 ± 0.90	PrfA*	78 ± 1.27
**0930**	**02029**	1	*yhfI*	wt	50 ± 1.43	PrfA*	37 ± 1.51
**0931**	**02030**	16	*lplA*	wt	28 ± 0.98	PrfA*	66 ± 0.92
**0964**	**02063**	1	*yjbH*	wt	56 ± 0.91	PrfA*	72 ± 1.19
**0980**	**02080**	1	*yadH*	wt	64 ± 1.61	PrfA*	73 ± 1.41
**1360**	**00810**	20	*folD*	wt	26 ± 1.15	PrfA*	32 ± 0.74
**1372**	**00822**	1	*acoA*	wt	51 ± 0.80	PrfA*	54 ± 0.85
**1490**	**00943**	4	*aroD*	wt	40 ± 1.41	PrfA*	37 ± 0.79
**1523**	**01447**	2	*relA*	wt	36 ± 0.89	PrfA*	42 ± 0.97
**1945**	**01092**	2	*ribU*	wt	54 ± 0.89	PrfA*	45 ± 1.19
**2049**	**01199**	1	hypothetical protein	wt	44 ± 0.85	PrfA*	39 ± 1.26
**2157**	**01675**	1	*sepA*	wt	76 ± 1.75	PrfA*	69 ± 1.39
**2168**	**01664**	2	*gloA*	wt	57 ± 0.73	PrfA*	104 ± 0.61
**2194**	**01638**	8	*oppC*	wt	18 ± 0.36	PrfA*	22 ± 0.57
**2195**	**01637**	2	*oppB*	wt	30 ± 1.22	PrfA*	39 ± 0.75
**2196**	**01636**	5	*oppA*	wt	29 ± 0.68	PrfA*	33 ± 1.41
**2215**	**01617**	1	ABC transporter ATP-binding protein	wt	70 ± 1.52	PrfA*	57 ± 1.05
**2250**	**01581**	1	*arpJ*	wt	33 ± 1.16	PrfA*	56 ± 0.77
**2386**	**02731**	1	*yuiD*	wt	51 ± 0.85	PrfA*	48 ± 0.90
**2448**	**01800**	1	SsrA-binding protein	wt	68 ± 1.07	PrfA*	74 ± 1.24
**2473**	**01775**	1	hypothetical protein	wt	52 ± 1.04	PrfA*	54 ± 1.06
**2474**	**01774**	1	hypothetical protein	wt	59 ± 1.54	PrfA*	54 ± 1.29
**2510**	**01738**	1	*secA* ^c^	wt	42 ± 1.17	PrfA*	47 ± 0.95
**2545**	**01702**	2	*thrB*	wt	42 ± 1.08	PrfA*	42 ± 1.47
**2546**	**01701**	1	*thrC*	wt	39 ± 1.29	PrfA*	37 ± 0.63
**2748**	**01948**	2	*ydaG* ^d^	wt	42 ± 0.92	PrfA*	36 ± 1.03
**2770**	**01925**	26	*gshF*	wt	39 ± 0.93	PrfA*	107 ± 1.91
**2843**	**01855**	1	hypothetical protein	wt	65 ± 1.31	PrfA*	61 ± 1.05
**0201–0202**	**02623–02624**	1	intergenic space	wt	42 ± 1.00	PrfA*	39 ± 0.85

^a^ Number of independent hits

^b^ Plaque size is an average of three independent replicates ± SEM (n = 40).

^c^ Transposon insertion was upstream *secA* gene.

^d^ Transposon insertion was upstream of the *ydaG* gene.

### Attenuated virulence of a *gloA* deletion mutant is fully rescued by PrfA*

**Fig 2:**
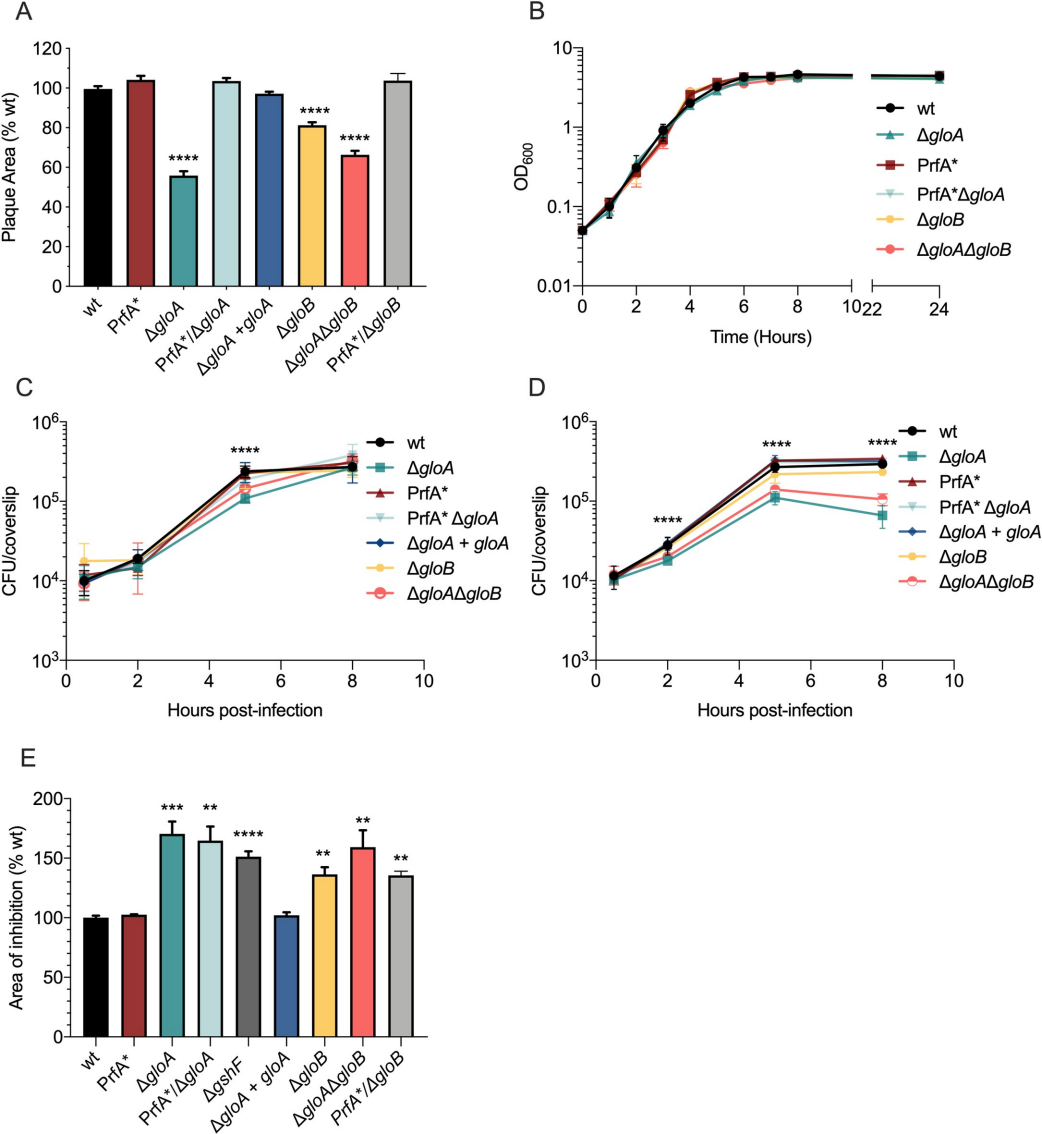
*In vitro* phenotypes of glyoxalase mutants. (A) Plaque area measured 3 days post-infection as a percentage of wild-type. Mean and standard error of the mean (SEM) pooled from three independent experiments is shown (n = 30). (B) Broth growth curve of indicated *L*. *monocytogenes* strains grown in BHI medium at 37°C with shaking. Mean and standard deviation of three independent experiments is shown. BMM’s were infected at an MOI of 0.25 with *L*. *monocytogenes* without treatment (C) or treated with PAM3CSK4 (D) and intracellular CFU were enumerated at different time points. Data are mean and SEM of three technical replicates of three independent experiments. For both panels, statistical significance is shown for Δ*gloA* compared to wild-type *L*. *monocytogenes*. (E) Sensitivity to MG (20% v/v) as measured by growth inhibition in a disk diffusion assay as percentage of wild-type. Data are mean and SEM of at least three independent experiments. For all experiments *p* values were calculated comparing to the wild-type bacteria using an unpaired Student’s *t*- test; *P < 0.05, **P < 0.01, ***P < 0.001, **** indicates P <0.0001.

**Fig 3:**
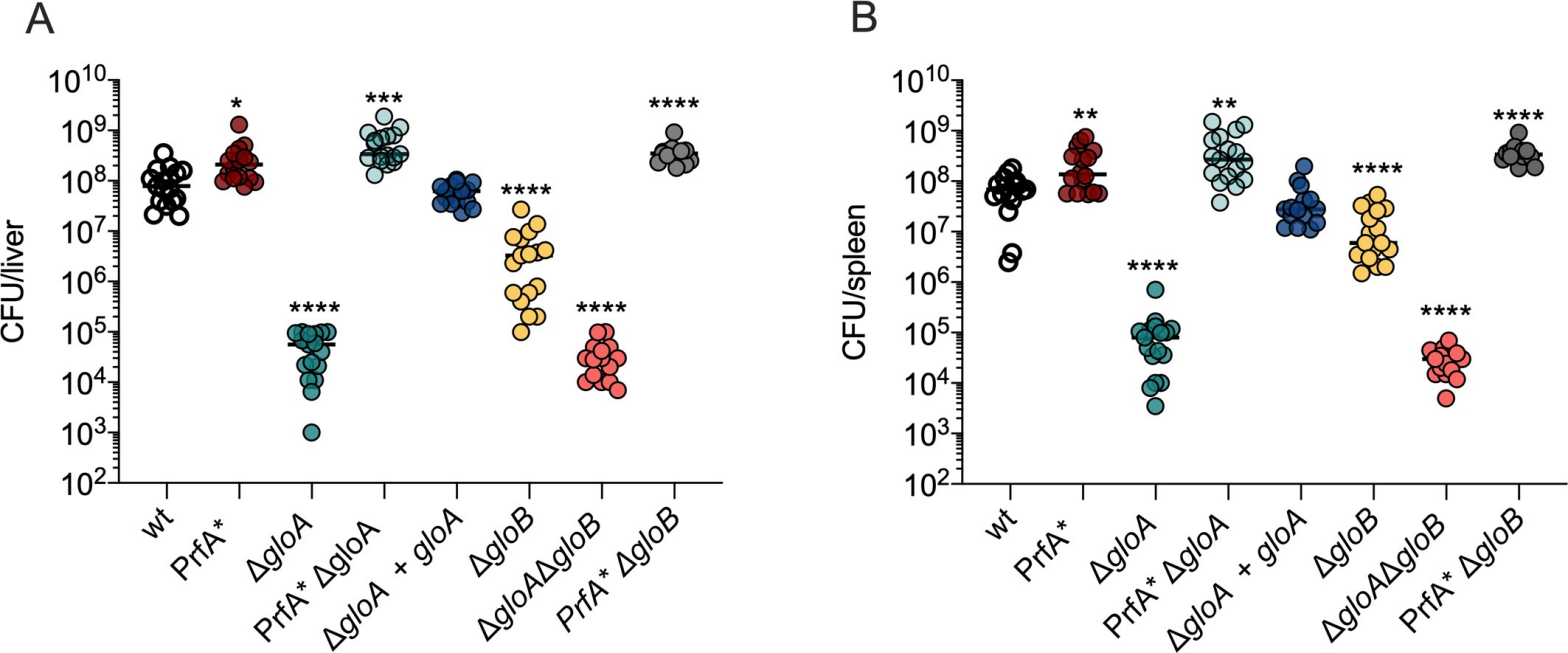
Virulence of glyoxalase mutants *in vivo*. (A and B) Female CD-1 mice were infected with 10^5^ CFU *L*. *monocytogenes*. Spleens (A) and livers (B) were harvested 48 hours post-infection and CFU were counted. Data and median represent three pooled experiments (n = 15). Data area mean and SEM of at least three independent experiments. For all experiments *p* values were calculated comparing to the wild-type bacteria using an unpaired Student’s *t*- test; *P < 0.05, **P < 0.01, ***P < 0.001, **** indicates P <0.0001.

To study the role of GloA-dependent MG detoxification in *L*. *monocytogenes* virulence activation, we generated an in-frame deletion of *gloA* in wild-type (Δ*gloA*) and PrfA* (PrfA*/Δ*gloA*) genetic backgrounds and characterized the mutants for growth and infection dynamics (Figs 2 and 3). The *gloA* deletion mutant had a 55% plaque size relative to wild-type that, similar to the *gloA* transposon mutant, was fully rescued in a PrfA* background. The plaque phenotype was also restored by complementation using an integration plasmid containing the *gloA* gene expressed from a *pHyper* promoter (Δ*gloA + gloA*; Fig 2A). Interestingly, the Δ*gloA* mutant had a negligible defect when cultured in broth and a small but significant difference at 5 hours-post infection of bone marrow-derived macrophage (BMM) when compared to wild-type *L*. *monocytogenes* (Fig 2B and 2C).

MG production is increased in toll-like receptor (TLR)-stimulated and IFN-γ activated macrophages [13,17]. We hypothesized that overnight treatment with PAM3CSK4, a TLR2 agonist, would stimulate BMMs and increase MG levels, affecting infection dynamics of Δ*gloA L*. *monocytogenes* that are impaired for MG detoxification. An intracellular growth defect was observed in the Δ*gloA* strain under these infection conditions, that was most evident at 8 hours post-infection and fully rescued by PrfA* (Fig 2D), suggesting that exogenous MG (i.e. produced by stimulated host cells) represented the primary source of this toxic metabolite during *L*. *monocytogenes* infection. To further assess the relevance of MG detoxification in *L*. *monocytogenes* virulence, CD-1 mice were infected intravenously with 10^5^ CFU of each strain and CFUs were determined in the spleens and livers harvested 48 hours post-infection. The Δ*gloA* mutant presented a 3-log virulence attenuation compared to wild-type *L*. *monocytogenes*. The virulence defect was fully rescued in a PrfA* background and complemented by insertion of an integrative plasmid containing the *gloA* gene expressed using a *pHyper* promoter (Fig 3).

Bacteria mainly use the glyoxalase system to detoxify MG, but may use alternative pathways as well [7,11]. To determine the relative contribution of the glyoxalase system in MG detoxification, we assessed *L*. *monocytogenes* strains for sensitivity against this toxic metabolite using a disk diffusion assay. The Δ*gloA* mutant was significantly more sensitive to MG than wild-type and PrfA* *L*. *monocytogenes* (Fig 2E). It is noteworthy that the PrfA* mutation did not rescue the Δ*gloA* strain to MG sensitivity, suggesting that the virulence defect could not be entirely attributed to MG toxicity. Sensitivity to MG in Δ*gloA* was fully complemented by insertion of the *gloA* gene expressed from a *phyper* promoter.

### Deletion of *gloB* results in attenuated virulence but to a lesser extent than *gloA*

The glyoxalase system is the major pathway used by cells for MG detoxification and is composed of two enzymes: glyoxalase A and B (Fig 1). A Δ*gloA* mutant was highly attenuated for virulence (Figs 2 and 3). To understand whether GloB was also critical for *L*. *monocytogenes* infection, we generated an in-frame deletion of the *gloB* gene and a double mutant that lacked both *gloA* and *gloB*. The Δ*gloB* mutant displayed a less attenuated virulence phenotype than Δ*gloA*, whereas the double mutant showed similar virulence defects (Figs 2 and 3). The plaque size of a Δ*gloB* mutant was approximately 80% relative to wild-type, while the Δ*gloA*Δ*gloB* double mutant was 65% of wild-type (Fig 2A). Both *L*. *monocytogenes* strains grew similar to wild-type in BMMs, but only the Δ*gloA*Δ*gloB* double mutant had a measurable defect for intracellular growth in PAM3CSK4-treated BMMs as observed for the Δ*gloA* mutant (Fig 2C–2D). The Δ*gloB* mutant had a 1-log virulence attenuation in murine infection and Δ*gloA*Δ*gloB* presented the same 3-log attenuation observed for the *gloA* single mutant (Fig 3). An increased sensitivity to MG when compared to wild-type *L*. *monocytogenes* was observed for all the glyoxalase mutants, but was less pronounced for Δ*gloB* (Fig 2E). Plaque size and mice attenuation of the Δ*gloB* mutant were fully rescued in a PrfA* background (Figs 2 and 3).

### Cytoplasmic acidification protects bacteria from methylglyoxal-induced mutagenesis

**Fig 4:**
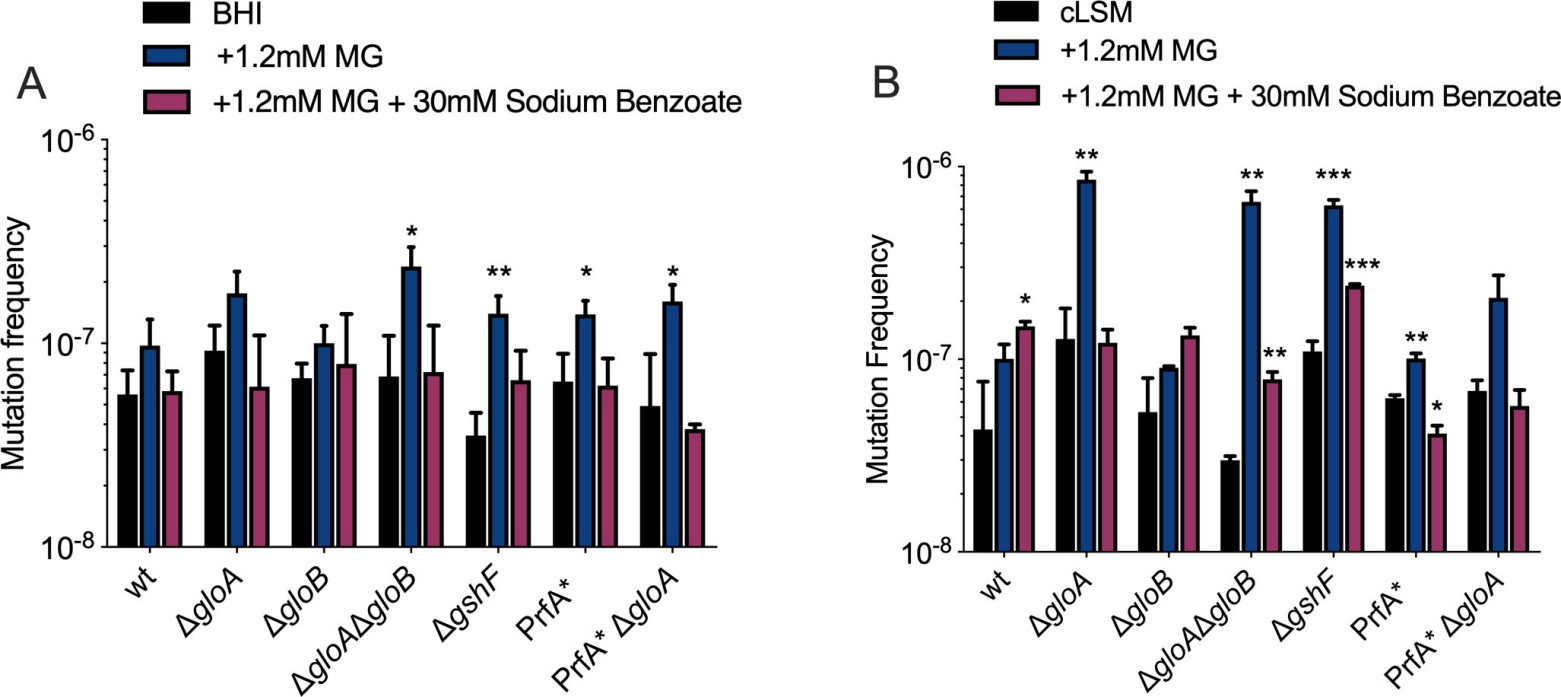
Mutation rates in *L*. *monocytogenes* glyoxalase mutants. Rifampicin mutation frequencies were determined in response to MG exposure and sodium benzoate. *L*. *monocytogenes* were grown overnight in BHI broth (A) or defined medium (cLSM) (B) and plated on BHI agar containing 5ug/mL of rifampicin. Mutation frequency was calculated as the ratio between the number CFU enumerated on the rifampicin plates and the total number of bacteria plated. Mean and SEM of two technical replicates from three independent experiments are shown. For all experiments *p* values were calculated comparing to the untreated bacteria using an unpaired Student’s *t*-test; *P < 0.05, **P < 0.01, ***P < 0.001, **** indicates P <0.0001.

MG is a highly reactive electrophile that interacts with DNA and has mutagenic effects [9,18,19]. To evaluate mutation rates as a result of MG exposure, we challenged *L*. *monocytogenes* strains with 1.2 mM MG in rich (BHI) and defined media (cLSM) and determined the frequency of rifampicin resistance. Mutation frequency consistently increased upon MG exposure compared to the non-treated groups in both growth conditions, but was more pronounced in defined media (Fig 4A and 4B). In defined media, mutants lacking *gloA* had at least an 8-fold increase in mutation frequency, that was also observed for the Δ*gshF* mutant but not for wild-type, PrfA* or Δ*gloB L*. *monocytogenes*. The PrfA* Δ*gloA* mutant had a lower mutation frequency compared to the Δ*gloA* mutant alone. Based on previous research [14], the Δ*gloB* mutant, unlike Δ*gloA*, should retain the capacity to activate potassium efflux pumps and acidify the bacterial cytoplasm leading to activation of a DNA damage response (Fig 1), and predictably, the *gloB* mutant had a lower mutation frequency.

Cytoplasmic acidification is sufficient for protection against MG in *B*. *subtilis* and *E*. *coli* most likely through the activation of a DNA damage response [10,13,14]. To assess if cytoplasmic acidification was sufficient to protect *L*. *monocytogenes* from MG-induced DNA damage, we determined rifampicin resistant derivatives upon MG exposure in media containing sodium benzoate, which decreases the pH of the bacterial cytoplasm by approximately 0.4 units and protects from MG-induced DNA damage [10,19]. Treatment with sodium benzoate decreased mutation frequency upon MG exposure to levels similar to the non-treated groups in both rich and defined media (Fig 4A and 4B), indicating that cytoplasmic acidification is protective against MG induced mutagenesis in *L*. *monocytogenes*.

### Methylglyoxal increases *gshF* mRNA but lowers glutathione availability in glyoxalase mutants

**Fig 5:**
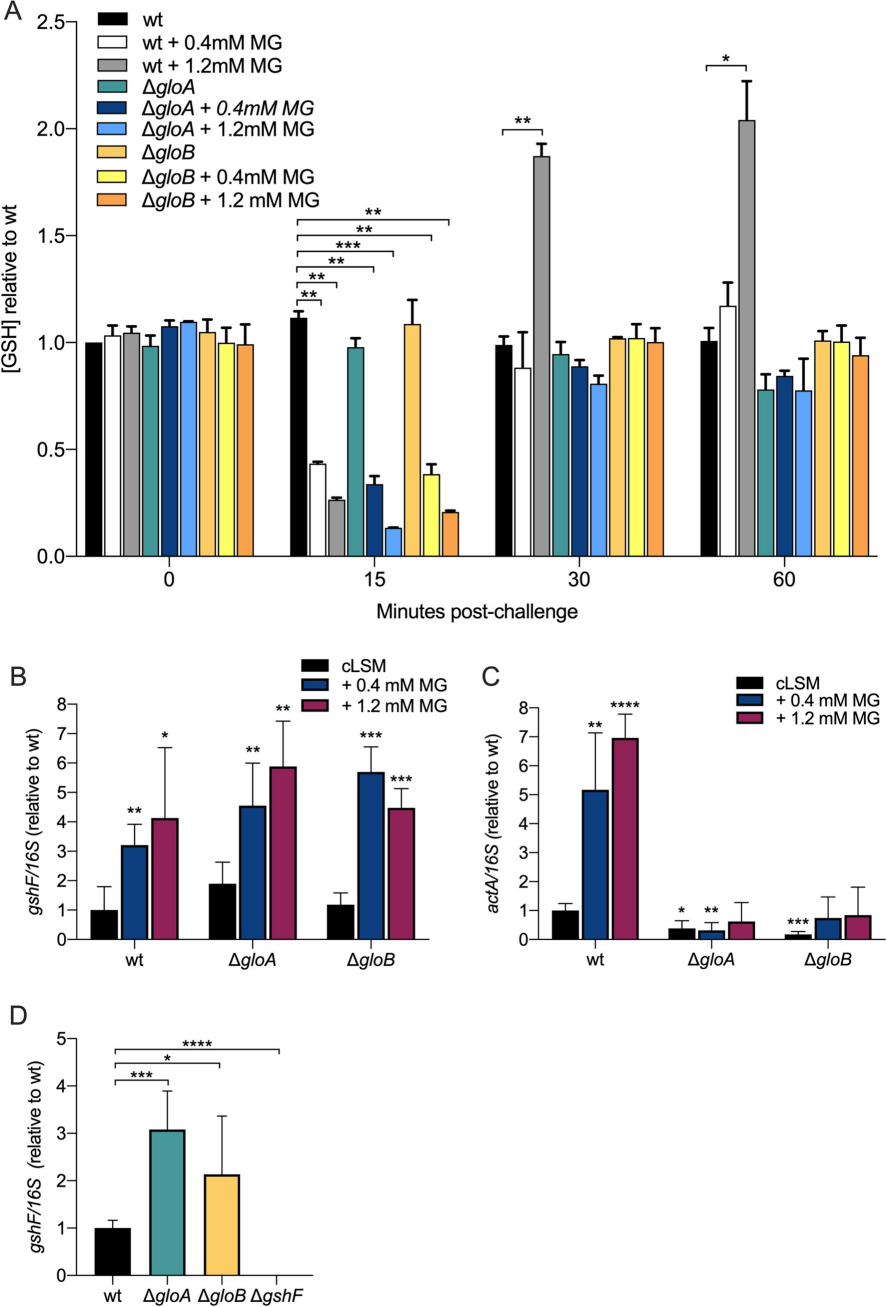
Glutathione, *gshF* mRNA and *actA* mRNA levels in glyoxalase mutants. (A) *L*. *monocytogenes* strains were grown to mid-log in defined media and challenged with 0.4 or 1.2 mM MG. Intracellular glutathione concentration was measured relative to wild-type at different time points post-challenge. Mean and SEM are shown of two technical replicates from two independent experiments. (B-C) Gene expression determined 15 minutes after MG exposure measured by RT-PCR. (D) Gene expression determined during BMMs infection measured by RT-PCR. Data are mean and SEM of two technical replicates from two independent experiments. For all experiments *p* values were calculated comparing to the wild-type bacteria using an unpaired Student’s *t*-test; *P < 0.05, **P < 0.01, ***P < 0.001, **** indicates P <0.0001.

The glyoxalase system detoxifies MG through two sequential enzymatic reactions. First, GSH and MG spontaneously form a hemithioacetal (Fig 1). Previous studies have shown that impairment in MG detoxification leads to decreased GSH availability in both mammalian and bacterial cells [7,10,14]. Since PrfA activation is dependent on the intracellular GSH levels, maintaining elevated concentrations is critical for *L*. *monocytogenes* to activate expression of virulence genes. We hypothesized that the glyoxalase mutants would have lower available intracellular levels of GSH, therefore, attenuated virulence. To test this hypothesis, we measured *L*. *monocytogenes* GSH concentration in wild-type, Δ*gloA* and Δ*gloB* strains at different time points post-MG exposure. We observed significantly lower available intracellular GSH at 15 minutes post-MG exposure in all strains (Fig 5A), however, by 30 minutes an increase in intracellular glutathione was observed. While the glyoxalase mutants reached GSH levels similar to the untreated wild-type *L*. *monocytogenes* at 30- and 60-minutes post-challenge, a significant increase was observed for the wild-type bacteria challenged with high amounts of MG (Fig 5A). These data indicated that although MG exposure immediately lowers available intracellular GSH in *L*. *monocytogenes*, it results in higher GSH concentration at later time points in the wild-type bacteria, but not in the glyoxalase mutants.

The capacity of bacteria to respond to redox stress is central for many cellular processes and survival. Antioxidants such as GSH are crucial for maintaining redox homeostasis. Previous studies have shown that upon oxidative stress, transcriptional regulators such as Spx in *B*. *subtilis* and CodY in *S*. *thermophilus* are induced and lead to upregulation of several genes including *gshF* and its analogue *bshA*, *B* and *C* [11,20,21]. We hypothesized that MG-induced oxidative stress functions as a metabolic cue that leads to upregulation of *gshF* expression in *L*. *monocytogenes*. To assess the expression of *gshF*, we performed real time-quantitative PCR to measure *gshF* mRNA levels upon MG exposure in defined media and during BMM infection. In defined media, *gshF* mRNA levels increased upon treatment with MG in all the *L*. *monocytogenes* strains tested, but was elevated in the *gloA* and *gloB* mutants (Fig 5B). During BMMs infection, *gshF* mRNA levels were also increased in the glyoxalase mutants compared to wild-type (Fig 5D). Consistent with the previous observations of increased *gshF* mRNA levels and higher intracellular GSH levels, we observed higher mRNA levels of *actA* upon MG challenge *in vitro* in wild-type *L*. *monocytogenes*, but not in the glyoxalase mutants (Fig 5C). Taken together, these data strongly suggest that MG toxicity induces expression of *L*. *monocytogenes* glutathione synthase and increases intracellular GSH as well.

## Discussion

The results of this study showed that *L*. *monocytogenes gloA* mutants are hyper-sensitive to exogenous MG toxicity *in vitro* and highly attenuated for virulence *in vivo*. Importantly the *in vivo* defect was fully rescued by a mutation in the master virulence regulator that locks it in its active conformation (PrfA*). These data suggest that the virulence defect of *gloA* mutants is at least partially due to lack of PrfA activation. Since PrfA is allosterically activated by GSH and GloA mediates MG detoxification by conjugation with glutathione, the results of this study suggest that *gloA* mutants are avirulent because of glutathione depletion and the consequent lack of PrfA activation. However, the PrfA* mutation did not rescue *L*. *monocytogenes* from MG lethality *in vitro* and the *gloB* deficient bacteria had less virulence attenuation than *gloA* mutants. These data suggest that some of the attenuation may be due to MG sensitivity. We also showed that MG initially depletes bacterial GSH levels and activates *gshF* gene expression, resulting in higher GSH levels for the wild-type bacteria but not for the glyoxalase mutants that are unable to recycle GSH used for MG detoxification. This suggests that MG is among the biological cues leading to glutathione production, PrfA activation and virulence.

The glyoxalase system is composed of two enzymes (GloA and GloB) that act sequentially to detoxify MG. The glyoxalase system is crucial for MG detoxification in *L*. *monocytogenes* as was evident by the significant increase in sensitivity to this toxic metabolite when either or both glyoxalase enzymes were missing. However, while the Δ*gloA* mutant was 3-logs less virulent in mice, the Δ*gloB* mutant was only 1-log less virulent. Analysis of mutation frequencies of the glyoxalase mutants revealed elevated mutation frequency in response to MG for Δ*gloA* but not for Δ*gloB*. Mutation rates were restored to wild-type levels in the Δ*gloA* mutant upon cytoplasmic acidification by addition of sodium benzoate a method used in previous studies [11,14]. Acidification of the cytosol is mediated by the enzymatic product of GloA, S-lactoylglutathione, which activates potassium efflux pumps and results in the exchange of protons that decrease cytoplasmic pH (see Fig 1). Acidification of the bacterial cytosol results in activation of DNA damage responses and probably has other protective effects as well [11,14,19]. In Δ*gloB* mutants, the formation of S-lactoylglutathione by GloA still occurs, leading to lower cytoplasmic pH and less DNA damage [14,15]. It is likely that the Δ*gloB* mutant was more tolerant to MG due to the functional activation of potassium efflux pumps and protective cytoplasmic acidification. Unlike the Δ*gloA* strain, which was probably killed by MG damage, protection by potassium efflux pumps allowed the Δ*gloB* strain to endure MG and activate alternative detoxification pathways [7,20,22]. However, since GloB converts S-lactoylglutathione to glutathione and D-lactate, the Δ*gloB* mutant also had less available glutathione (Fig 5A) which likely leads to less PrfA activation and attenuated virulence.

It is hard to reconcile why a PrfA* background fully rescued the Δ*gloA* mutant given that the Δ*gloB* mutant had the same GSH levels, at least *in vitro*, suggesting that virulence attenuation is not entirely due to GSH depletion. Notably, while the PrfA* Δ*gloA* mutant had the same sensitivity to MG as the Δ*gloA* strain by disk diffusion, it had a lower MG-induced mutation frequency than the Δ*gloA* mutant. We hypothesize that PrfA activation protects the Δ*gloA* mutant from MG-induced DNA damage, although the mechanism and genes involved await further exploration. If our hypothesis is correct, it suggests that MG leads to PrfA activation and that activated PrfA not only mediates expression of the known virulence genes, but also mediates the bacterial response to MG toxicity and perhaps other host-derived stressors. Our lab continues to explore MG-induced DNA-damage and the possible roles played by PrfA activation.

MG is a RES that causes oxidative stress through increased generation of ROS and protein glycation [23–25]. *L*. *monocytogenes* produces this toxic byproduct of metabolism; thus, it must protect itself from MG produced by its cellular processes and that of its host. The fact that the glyoxalase mutants grew normally in broth but had a defect for growth in TLR-activated macrophages and in mice, suggests that host-derived MG imposes the major threat to bacteria during its intracellular growth, although we cannot rule out that changes in *L*. *monocytogenes* metabolism may also play a role. TLRs are pattern recognition receptors that upon stimulation, orchestrate an immune response by activating multiple signaling pathways that result in a variety of genetic and metabolic changes [25], including increased glycolysis leading to increases in MG [17,26,27]. Activated macrophages not only increase MG production, they also down-regulate expression of their own glyoxalase enzymes, which may contribute to the inflammatory response [28] and may suggest a role for MG as an antimicrobial strategy.

Upon infection, *L*. *monocytogenes* faces numerous stressors. The most striking finding of our study is that upon MG stress *in vitro*, *gshF* transcription is activated, suggesting that MG is one of the metabolic and redox cues sensed by this pathogen *in vivo*. Accordingly, glyoxalase mutants had higher levels of *gshF* mRNA upon infection compared to wild-type *L*. *monocytogenes*. Our working model is that *L*. *monocytogenes* encounters MG in high quantities in the host cytosol and that stress caused by MG, and probably other ROS and RES, activates transcriptional regulators such as Spx that induce *gshF* transcription [11,21]. Indeed, a recent study reported that in *L*. *monocytogenes*, SpxA1 activated *gshF* transcription [27]. The results reported in this study suggest that RES and ROS encountered in the cytosol might be the host cues recognized by this pathogen to sense their entry into the host cell. Whether MG is the most prevalent RES and redox stress experienced in the cytosol of mammalian cells remains to be determined. Further studies on redox cues by which intracellular bacteria sense their host will help better understand how bacteria sense their environment and regulate virulence genes in response.

## Methods

### Ethics statement

All animal work was done in strict accordance with the recommendations in the Guide for the Care and Use of Laboratory Animals of the National Institutes of Health and university regulations. Protocols were reviewed and approved by the Animal Care and Use Committee at the University of California, Berkeley **AUP 2016-05-8811**.

### Bacterial cultures and strains

The parental strain for all *L*. *monocytogenes* strains used in this study **(**S1 Table) is 10403S. Bacteria were cultivated overnight at 37°C shaking in Brain-Heart Infusion (BHI; BD) with streptomycin (GoldBio) unless otherwise stated. All *E*. *coli* strains (S2 Table) were grown in Luria broth (LB) at 37°C shaking. Antibiotics were used at the following concentrations: streptomycin (200 μg/mL), erythromycin (1 μg/mL), carbenicillin (100 μg/mL) and chloramphenicol (7.5 μg/mL for *L*. *monocytogenes;* 10 μg/mL for *E*. *coli*). For broth growth curves, overnight cultures were diluted in 35 mL of fresh BHI to an initial optical density at 600 nm (OD_600_) of 0.05. Bacteria were cultured at 37°C with shaking and growth was measured spectrophotometrically every hour [29].

For the generation of transposon libraries, electro-competent *L*. *monocytogenes* were prepared, and *himar-1* transposon mutagenesis was performed as described previously [30]. Transposon insertion sites were identified [30] and mapped to the 10403S genome. Transposons in *L*. *monocytogenes* were introduced into a wild-type and PrfA* background by transduction using the phage U153 as described previously [31]. *Himar-1* transposon transduction was selected on erythromycin (Sigma-Aldrich) after two days. Single colonies were sequenced for verification.

### Cloning and plasmid construction

Deletions of genes were performed by allelic exchange using primers listed in S3 Table and the pKSV7 plasmid [32]. Briefly, constructed pKSV7 plasmids were transformed into XL1 Blue and SM10 *E*. *coli*, recovered on LB agar plates containing carbenicillin (Sigma-Aldrich) and conjugated into *L*. *monocytogenes* on non-selective BHI agar. *L*. *monocytogenes* carrying the knock-out plasmid were selected on BHI containing streptomycin and chloramphenicol at 30°C and re-streaked at 42°C three consecutive times on BHI containing the same antibiotics to select for chromosomal integration. This selected strain was serially passaged at 30°C shaking to facilitate loss of pKSV7. Mutants that lost the plasmid were identified by patch-plating methods and confirmed by PCR and Sanger sequencing [33]. The *gloA* complementation vector was constructed as previously described [34] in a pPL2 plasmid using a Phyper promoter. The plasmid was transformed into XL-Blue and SM10 *E*. *coli*. Following confirmation by Sanger sequencing, it was conjugated into wild-type and Δ*gloA L*. *monocytogenes*.

### Plaque screen and assay

The mouse L2 fibroblast cell line was cultured in Dulbecco’s Modified Eagle Medium (DMEM; Gibco) supplemented with 10% Fetal Bovine Serum (FBS; Seradigm), 1% sodium pyruvate, 1% L- glutamine and 1% penicillin-streptomycin (Corning). L2 cells were grown in T75 flasks at 37°C and split once cells were confluent (every 3–4 days). In all experiments, L2 cells were plated overnight so that they were confluent the day of the infection. A *himar-1* transposon library composed of approximately 10,000 *L*. *monocytogenes* mutants [30] was used to screen for transposon insertions that caused a small-plaque phenotype. For the initial screening for small-plaque mutants, 4 x 10^6^ L2 cells were seeded in 100-mm petri dishes and infected at an MOI of 0.1. The plaque assay was performed as previously described [16]. L2 cells are commonly used to perform this particular assay because they generate highly homogenous and reproducible plaques [16]. Plaques were imaged 72 hours post-infection and small plaques were selected. Selected small plaque mutants were purified by repeating the plaque-formation assay until the plaque phenotype was completely homogenous. Transposon mutants were sequenced and transduced intro different genetic backgrounds. For routine plaque assays, six-well plates were seeded with 1.2 x 10^6^ L2 cells per well and infected at an MOI of 0.1. The plaque assay was performed as described previously [16]. Plaques were imaged 72 hours post-infection and plaque area was quantified using ImageJ software. Each experiment represents an average of the area of ten plaques per strain as a percentage of wild-type *L*. *monocytogenes*. Groups were statistically compared using an unpaired Student’s *t*-test.

### Intracellular growth curves

Macrophage growth curves were performed as previously described [35]. Bone marrow derived macrophages (BMMs) were derived from bone marrow of C57BL/6 mice (Jackson Laboratory) and cultivated in DMEM medium containing 10% CSF (from M-CSF-producing 3T3 cells), 20% FBS, 1% L-glutamine, 1% sodium pyruvate and 14 mM 2-mercaptoethanol (Gibco). A total of 3 x 10^6^ BMMs were plated in 60 mm petri dishes containing 14 12 mm glass coverslips in each dish. For indicated experiments, BMMs were seeded overnight with medium containing PAM3CSK4 (Invivogen) at a final concentration of 100ng/mL. These dishes were infected the next day at an MOI of 0.25 for 30 minutes, washed twice with sterile PBS and 50 μg/mL gentamicin (Sigma-Aldrich) was added 1-hour post-infection. Three coverslips were removed at each time point, rigorously mixed in sterile water and plated on LB agar with streptomycin. Each experiment represents the average of three coverslips per time point per strain.

### Virulence in mice

Eight-week-old female CD-1 mice (Charles River Laboratories) were infected intravenously via the tail vein with 1 x 10^5^ CFU of *L*. *monocytogenes* strains in 200 μL of sterile PBS as described [31]. Forty-eight hours post-infection, mice were euthanized, and spleens and livers were harvested, homogenized in 0.1% NP-40 (Sigma-Aldrich) in water, and plated on LB agar with streptomycin. Groups were statistically compared using an unpaired Student’s *t*-test.

### Disk-diffusion assay

Disk-diffusion assays were performed similarly to methods described previously [31,33]. 1 x 10^6^ CFUs from overnight cultures grown at 37°C shaking were mixed in 4 mL of top agar (0.8% NaCl and 0.8% bacto-agar) and spread evenly on BHI plates containing streptomycin. Whatman paper disks containing 20 μL of 20% MG (Sigma-Aldrich) were placed on top of the cooled agar. The zone of inhibition was measured 18–24 hours after incubation at 37°C. Total inhibition area as percentage of wild-type *L*. *monocytogenes* is presented from at least three independent experiments. Statistical significance was determined using an unpaired Student’s *t*-test.

### Rifampicin mutagenesis assay

Mutation frequency in rifampicin was determined using similar previously described methods [36]. *L*. *monocytogenes* strains were grown at 37°C shaking in fresh BHI or defined media (cLSM) [37], containing 1.2 mM MG or 1. 2mM MG plus 30 mM Sodium benzoate (Sigma-Aldrich). The next day, OD_600_ was measured for each strain and 100 μL of overnight cultures were plated in LB agar plates containing 5 μg/mL of rifampicin (Sigma-Aldrich). CFUs were counted after a 24-hour incubation at 37°C. Mutation frequency was calculated as the ratio between CFUs enumerated in the LB agar plates containing rifampicin and the total number of *L*. *monocytogenes* plated. Data represents the average of two technical replicates from three independent experiments.

### Glutathione assay

Reduced glutathione (GSH) concentrations were measured by using a commercial kit supplied by Sigma-Aldrich (CS0260) according to the manufacturer’s specifications. Briefly, overnight *L*. *monocytogenes* cultures were diluted to an OD_600_ of 0.1 in 35 mL of fresh defined media and grown at 37°C shaking. After two hours, 10 OD_600_ were transferred to 15 mL Falcon tube and 0.4 or 1.2 mM of MG was added to the indicated cultures. The media was supplemented with cysteine doubling the amount of MG added (0.8 mM or 2.2 mM) 15 minutes after the addition of MG. For the untreated cultures a total of 0.8 mM cysteine was added. Bacteria in the Falcon tube were washed twice in sterile PBS and resuspended in 200 μL of 5% 5-Sulfosalicylic Acid. Bacteria were lysed 0.1 mm-diameter silica-zirconium beads and 10 μL were used for the kit’s working reaction. Samples were taken at 15, 30 and 60 minutes post-challenge with MG. Absorbance at 412 nm was measured using a plate reader (Infinite M1000 PRO, TECAN). GSH concentrations as a percentage of wild-type without MG challenge are an average of two technical replicates from three independent experiments.

### Quantitative RT-PCR of bacterial transcripts

Transcript analysis in defined media was performed as described previously [38]. Overnight cultures were diluted 1:10 in 5 mL of fresh cLSM. When the culture reached mid-log growth, 0.4 mM or 1.2 mM MG were added to the cultures. *L*. *monocytogenes* strains were harvested 15 minutes post-MG addition at an OD_600_ of 0.5. Transcript analysis during infection was performed as previously described [5]. Briefly, 3x10^7^ BMMs were plated in 150 mm TC-treated dishes and infected with an MOI of 10. One-hour post-infection the cells were washed with PBS and media containing gentamicin (50 μg/mL) was added. Four hours post-infection the cells were washed with PBS and lysed in 5 mL of 0.1% NP-40. RNAprotect Bacteria Reagent (Qiagen) was used to rinse the dishes, which was combined with the lysate. Bacteria were collected by centrifugation. RNA from bacteria harvested from either cLSM or BMMs was obtained using Quick RNA fungal/bacterial Miniprep from Zymo Research (R2014). Obtained nucleic acids were treated with TURBO DNase (Invitrogen) and concentrated with the RNA clean and concentrator kit from Zymo Research (R2017). RNA was reverse transcribed with iScript (Bio-RAD) and quantitative PCR (qPCR) was performed using SYBR FAST (Kapa Biosystems). Primers used for qPCR are listed in S3 Table.

### Statistical analysis

Data were analyzed using GraphPad Prism 8. * indicates P <0.05; ** indicates P <0.01, *** indicates P <0.001, **** indicates P <0.0001.

## Supporting information

S1 Table *L. monocytogenes* strains used in this study. (DOCX)

S2 Table *E. coli* strains used in this study. (DOCX)

S3 Table Oligonucleotide primers used in this study. (DOCX)
