# Supplementary material for: Detoxification of methylglyoxal by the glyoxalase system is required for glutathione availability and virulence activation in Listeria monocytogenes
Source: PLoS Pathog. 2021 Aug 18;17(8):e1009819. doi: 10.1371/journal.ppat.1009819 (PMC8372916; doi:10.1371/journal.ppat.1009819)
Supplement: S1 Table — (DOCX) [file ppat.1009819.s001.docx]

S1 Table. *L. monocytogenes* strains used in this study.

| **Strain name** | **Description** | **Reference** |
| --- | --- | --- |
| 10403S | wt | [1] |
| DP-L5451 | PrfA* | [2] |
| DP-L7263 | *plcA::Tn (lmo0201)* | This study |
| DP-L7264 | *hly::Tn (lmo0202)* | This study |
| DP-L7265 | *mpl::Tn (lmo0203)* | This study |
| DP-L7266 | *plcB::Tn (lmo0205)* | This study |
| DP-L7267 | *lmo0402::Tn* | This study |
| DP-L7268 | *lmo0898::Tn* | This study |
| DP-L7269 | *lmo0930::Tn* | This study |
| DP-L7270 | *lplA::Tn (lmo0931)* | This study |
| DP-L7271 | *yjbH::Tn (lmo0964)* | This study |
| DP-L7272 | *lmo0980::Tn* | This study |
| DP-L7273 | *folD::Tn (lmo1360)* | This study |
| DP-L7274 | *lmo1372::Tn* | This study |
| DP-L7275 | *lmo1424::Tn* | This study |
| DP-L7276 | *aroD::Tn (lmo1490)* | This study |
| DP-L7277 | *relA::Tn (lmo1523)* | This study |
| DP-L7278 | *ribU::Tn (lmo1945)* | This study |
| DP-L7279 | *lmo2049::Tn* | This study |
| DP-L7280 | *lmo2157::Tn* | This study |
| DP-L7281 | *gloA::Tn (lmo2168)* | This study |
| DP-L7282 | *oppC::Tn (lmo2194)* | This study |
| DP-L7283 | *oppB::Tn (lmo2195)* | This study |
| DP-L7284 | *oppA::Tn (lmo2196)* | This study |
| DP-L7285 | *lmo2215::Tn* | This study |
| DP-L7286 | *aprJ::Tn (lmo2250)* | This study |
| DP-L7287 | *yuiD::Tn (lmo2386)* | This study |
| DP-L7288 | *lmo2448::Tn* | This study |
| DP-L7289 | *lmo2473::Tn* | This study |
| DP-L7290 | *lmo2474::Tn* | This study |
| DP-L7291 | *lmo2510::Tn* | This study |
| DP-L7292 | *thrB::Tn (lmo2545)* | This study |
| DP-L7293 | *thrC::Tn (lmo2546)* | This study |
| DP-L7294 | *ydaG::Tn (lmo2748)* | This study |
| DP-L7295 | *ghsF::Tn (lmo2770)* | This study |
| DP-L7296 | *lmo2843::Tn* | This study |
| DP-L7297 | *lmo201-0202::Tn* | This study |
| DP-L7298 | PrfA* *plcA::Tn (lmo0201)* | This study |
| DP-L7299 | PrfA* *hly::Tn (lmo0202)* | This study |
| DP-L7300 | PrfA* *mpl::Tn (lmo0203)* | This study |
| DP-L7301 | PrfA* *plcB::Tn (lmo0205)* | This study |
| DP-L7302 | PrfA* *lmo0402::Tn* | This study |
| DP-L7303 | PrfA** lmo0898::Tn* | This study |
| DP-L7304 | PrfA* *lmo0930::Tn* | This study |
| DP-L7305 | lPrfA* *lplA::Tn (lmo0931)* | This study |
| DP-L7306 | PrfA* *yjbH::Tn (lmo0964)* | This study |
| DP-L7307 | lPrfA* *lmo0980::Tn* | This study |
| DP-L7308 | PrfA* *folD::Tn (lmo1360)* | This study |
| DP-L7309 | lPrfA* *lmo1372::Tn* | This study |
| DP-L7310 | PrfA* *lmo1424::Tn* | This study |
| DP-L7311 | PrfA* *aroD::Tn (lmo1490)* | This study |
| DP-L7312 | PrfA* *relA::Tn (lmo1523)* | This study |
| DP-L7313 | PrfA* *ribU::Tn (lmo1945)* | This study |
| DP-L7314 | PrfA** lmo2049::Tn* | This study |
| DP-L7315 | PrfA* *lmo2157::Tn* | This study |
| DP-L7316 | PrfA* *gloA::Tn (lmo2168)* | This study |
| DP-L7317 | PrfA* *oppC::Tn (lmo2194*) | This study |
| DP-L7318 | PrfA* *oppB::Tn (lmo2195)* | This study |
| DP-L7319 | PrfA* *oppA::Tn (lmo2196)* | This study |
| DP-L7320 | PrfA* *lmo2215::Tn* | This study |
| DP-L7321 | PrfA* *aprJ::Tn (lmo2250)* | This study |
| DP-L7322 | PrfA* *yuiD::Tn (lmo2386)* | This study |
| DP-L7323 | PrfA** lmo2448::Tn* | This study |
| DP-L7324 | PrfA* *lmo2473::Tn* | This study |
| DP-L7325 | PrfA* *lmo2474::Tn* | This study |
| DP-L7326 | PrfA* *lmo2510::Tn* | This study |
| DP-L7327 | PrfA* *thrB::Tn (lmo2545)* | This study |
| DP-L7328 | PrfA* *thrC::Tn (lmo2546)* | This study |
| DP-L7329 | PrfA* *ydaG::Tn (lmo2748)* | This study |
| DP-L7330 | PrfA* *ghsF::Tn (lmo2770)* | This study |
| DP-L7331 | PrfA* *lmo2843::Tn* | This study |
| DP-L7332 | PrfA* *lmo201-0202::Tn* | This study |
| DP-L6188 | Δ*gshF* | [3] |
| DP-L7333 | Δ*gloA (lmo2168)* | This study |
| DP-L7334 | Δ*gloB (lmo2167)* | This study |
| DP-L7335 | Δ*gloA*Δ*gloB* | This study |
| DP-L7336 | PrfA* Δ*gloA* | This study |
| DP-L7337 | Δ*gloA* pPL2.*phyper.gloA* | This study |

**References**

1. Bécavin C, Bouchier C, Lechat P, Archambaud C, Creno S, Gouin E, et al. Comparison of Widely Used Listeria monocytogenes Strains EGD, 10403S, and EGD-e Highlights Genomic Differences Underlying Variations in Pathogenicity. Casadevall A, editor. MBio [Internet]. 2014 Mar 25;5(2). Available from: https://mbio.asm.org/lookup/doi/10.1128/mBio.00969-14

2. Miner MD, Port GC, Freitag NE. Functional impact of mutational activation on the Listeria monocytogenes central virulence regulator PrfA. Microbiology [Internet]. 2008 Nov 1;154(11):3579–89. Available from: https://www.microbiologyresearch.org/content/journal/micro/10.1099/mic.0.2008/021063-0

3. Reniere ML, Whiteley AT, Hamilton KL, John SM, Lauer P, Brennan RG, et al. Glutathione activates virulence gene expression of an intracellular pathogen. Nature [Internet]. 2015 Jan 8;517(7533):170–3. Available from: http://www.ncbi.nlm.nih.gov/pubmed/25567281
