# Supplementary material for: Detoxification of methylglyoxal by the glyoxalase system is required for glutathione availability and virulence activation in Listeria monocytogenes
Source: PLoS Pathog. 2021 Aug 18;17(8):e1009819. doi: 10.1371/journal.ppat.1009819 (PMC8372916; doi:10.1371/journal.ppat.1009819)
Supplement: S2 Table — (DOCX) [file ppat.1009819.s002.docx]

S2 Table. *E. coli* strains used in this study

| **Strain name** | **Description** | **Reference** |
| --- | --- | --- |
| XL 1 Blue | For vector construction | Stratagene |
| SM10 | For conjugation | [1] |
| DP-L7338 | pKSV7.Δ*gloA* | This study |
| DP-L7339 | pKSV7.Δ*gloB* | This study |
| DP-L7340 | pPL2.*phyper.gloA* | This study |

**Reference**

1. Simon R, Priefer U, Pühler A. A Broad Host Range Mobilization System for In Vivo Genetic Engineering: Transposon Mutagenesis in Gram Negative Bacteria. Bio/Technology [Internet]. 1983 Nov;1(9):784–91. Available from: http://www.nature.com/doifinder/10.1038/nbt1183-784
