# Supplementary material for: Detoxification of methylglyoxal by the glyoxalase system is required for glutathione availability and virulence activation in Listeria monocytogenes
Source: PLoS Pathog. 2021 Aug 18;17(8):e1009819. doi: 10.1371/journal.ppat.1009819 (PMC8372916; doi:10.1371/journal.ppat.1009819)
Supplement: S3 Table — (DOCX) [file ppat.1009819.s003.docx]

S3 Table. Oligonucleotide primers used in this study.

| **Target gene** | **Forward primer sequence*** | **Reverse primer sequence*** |
| --- | --- | --- |
| pKSV7 | gctgcaggaggcagtgga | ggatccagcgccgct |
| pKSV7.ΔgloA 5' | atggggtccagcggcgctggatcctgcgcagtacagactcttctgg | tgtatgtaacatttttgcagtcatttttattcctcc |
| pKSV7.ΔgloA 3' | ctgcaaaaatgttacatacacaagatggcgctttataagtagaagaaggac | gctcgctccactgcctcctgcagccagcagacacattacgagaacaaattcg |
| pKSV7.ΔgloB 5' | atggggtccagcggcgctggatcccttggcgagagtaattgagcacc | cgtttgatttttgtcggtcatgaaaagaagacaaacccataccggtcatgaaaagaagacaaacccatac |
| pKSV7.ΔgloB 3' | cgtttgatttttgtcatttcacttccg | gctcgctccactgcctcctgcagccggtacttgacgcaacggatac |
| pPL2.phyper.gloA | gtaaaccttaaactgcatgtcgactcacttccgcctttcttcacattca | ggatcccatcatcatcatcatcacagtgcctgtaatgatgacagcgataaggcttg |
| 16S rRNA | acccttgattttagttgccag | tgtgtagcccaggtcataag |
| gshF rRNA | gaccctaatctccggaagc | tacagagtcaatcgagtccg |
| actA rRNA | cgacataatatttgcagcgac | tgctttcaacattgctattagg |

*Oligonucleotide primers listed 5’-3’ indicate restriction or complementary sited for Gibson Assembly.
